# Prognostic Value of Mitotic Index and Bcl2 Expression in Male Breast Cancer

**DOI:** 10.1371/journal.pone.0060138

**Published:** 2013-04-01

**Authors:** Miangela M. Lacle, Carmen van der Pol, Arjen Witkamp, Elsken van der Wall, Paul J. van Diest

**Affiliations:** 1 Department of Pathology, University Medical Center Utrecht, Utrecht, The Netherlands; 2 Department of Surgery, University Medical Center Utrecht, Utrecht, The Netherlands; 3 Division of Internal Medicine and Dermatology, University Medical Center Utrecht, Utrecht, The Netherlands; Health Canada, Canada

## Abstract

The incidence of male breast cancer (MBC) is rising. Current treatment regimens for MBC are extrapolated from female breast cancer (FBC), based on the assumption that FBC prognostic features and therapeutic targets can be extrapolated to MBC. However, there is yet little evidence that prognostic features that have been developed and established in FBC are applicable to MBC as well. In a recent study on FBC, a combination of mitotic index and Bcl2 expression proved to be of strong prognostic value. Previous papers on Bcl2 expression in MBC were equivocal, and the prognostic value of Bcl2 combined with mitotic index has not been studied in MBC. The aim of the present study was therefore to investigate the prognostic value of Bcl2 in combination with mitotic index in MBC. Immunohistochemical staining for Bcl2 was performed on tissue microarrays of a total of 151 male breast cancer cases. Mitotic index was scored. The prognostic value of Bcl2 expression and Bcl2/mitotic index combinations was evaluated studying their correlations with clinicopathologic features and their prediction of survival. The vast majority of MBC (94%) showed Bcl2 expression, more frequently than previously described for FBC. Bcl2 expression had no significant associations with clinicopathologic features such as tumor size, mitotic count and grade. In univariate survival analysis, Bcl2 had no prognostic value, and showed no additional prognostic value to tumor size and histological grade in Cox regression. In addition, the Bcl2/mitotic index combination as opposed to FBC did not predict survival in MBC. In conclusion, Bcl2 expression is common in MBC, but is not associated with major clinicopathologic features and, in contrast to FBC, does not seem to have prognostic value, also when combined with mitotic index.

## Introduction

The incidence of male breast cancer (MBC) is relatively low compared to female breast cancer (FBC) but is rising [1]. Currently, treatment regimens for MBC are based on the assumption that it is similar to its female counterpart, and that FBC prognostic features and therapeutic targets can be extrapolated to MBC. Although there are indeed similarities between MBC and FBC, there is also mounting evidence that they are in fact biologically quite different [2]–[7]. At the same time, there is yet little evidence that prognostic features that have been developed and established in FBC work on MBC as well.

Cellular proliferation is a strong traditional prognostic feature in FBC [8], [9] and was shown to have important prognostic value in MBC as well already years ago [10]. Cellular proliferation can be assessed in different ways, but counting mitoses is probably the most widely studied and widely applied way of assessing cellular proliferation [8], [9], and is the main prognostic constituent of histological grading [11].

In a recent study on FBC, a combination of mitotic index and Bcl2 expression proved to be of value in stratifying patients into low risk vs. high risk groups regarding mortality and recurrence [12]. Bcl2 is an anti-apoptotic protein which is significantly more often expressed in male breast cancer compared to FBC [13], [14], [15]. Although there are some data published regarding Bcl2 expression and its prognostic value in MBC, the prognostic value of Bcl2 combined with mitotic index has not been studied in MBC. The aim of the present study was therefore to investigate the prognostic value of Bcl2 in combination with mitotic index in MBC and evaluate whether its prognostic value equals that observed in FBC.

## Materials and Methods

### 2.1 Ethics Statement

Since we used archival pathology material which does not interfere with patient care and does not involve the physical involvement of the patient, no ethical approval is required according to Dutch legislation [the Medical Research Involving Human Subjects Act (Wet medisch-wetenschappelijk onderzoek met mensen, WMO [16])]. Use of anonymous or coded left over material for scientific purposes is part of the standard treatment contract with patients and therefore informed consent procedure was not required according to our institutional medical ethical review board. This has also been described by van Diest et al. [17].

### 2.2 Patients and Specimens

All consecutive cases of surgical breast specimens of invasive male breast cancer from 1986–2011 were collected from 7 different pathology laboratories in The Netherlands (St. Antonius Hospital Nieuwegein, Diakonessenhuis Utrecht, University Medical Center Utrecht, Laboratory for Pathology East Netherlands) and in Germany (Paderborn, Koeln, Kassel) as described before [4]–[7]. Hematoxylin and eosin (HE) slides were reviewed by three experienced observers (PJvD, RK, MML) to confirm the diagnosis and assess tumor characteristics. The tumors were categorized by histological type according to the WHO [18]. Pathology reports were used to retrieve information on age, tumor size and lymph node status. A total of 151 cases were included from which there was enough material left in the paraffin blocks to perform immunohistochemistry. Table 1 shows baseline clinicopathological data.

**Table 1:** Baseline clinicopathologic features of 151 male breast cancers.

Characteristics	All cases (n = 151)	Characteristics	All cases (n = 151)
Age (mean), years	66 (n = 150)	Histological grade	
</ = 50	15 (10%)	I	38 (25.2%)
>50	135 (90%)	II	66 (43.7%)
		III	47 (31.1%)
Histological type			
Ductal	136 (90%)	Lymph node metastasis	n = 124
Lobular	3 (2%)	Absent	56 (45.2%)
Invasive cribriform	3 (2%)	Present	68 (54.8%)
Mixed (ductal/lobular)	3 (2%)		
Mucinous	2 (1.3%)	Immunohistochemistry	
Papillary	2 (1.3%)	ER	n = 150
Invasive micropapillary	1 (0.7%)	(+)	135 (90%)
Adenoid cystic	1 (0.7%)	(−)	15 (10%)
Tumor size (mean), cm	2.235 (n = 147)	PR	n = 150
T1	79 (54.1)	(+)	99 (66%)
T2	64 (43.5%)	(−)	51 (34%)
T3	4 (2.7%)		
		AR	n = 150
Tubule formation		(+)	120 (80%)
>75%	14 (9.3%)	(−)	30 (20%)
10–75%	59 (39.1%)		
<10%	78 (51.7%)	HER2	n = 150
		(+)	5 (3.3%)
Nuclear atypia		(−)	145 (96.7%)
Mild	13 (8.6%)		
Moderate	89 (58.9%)	Bcl2 (10%)	n = 149
Severe	49 (32.5%)	(+)	140 (94%)
		(−)	9 (6%)
Mitotic activity index/2 mm^2^			
<8 mitoses	60 (49.7%)	Bcl2 (30%)	n = 149
8–14 mitoses	40 (26.5%)	(+)	137 (92%)
15 or>mitoses	51 (33.8%)	(−)	12 (8%)

### 2.3 Mitosis Counting

Mitotic activity was assessed on regular sections according to the protocol described earlier [19], [20] and expressed per 2 mm^2^. Mitotic index was scored as follows: M1 =  low if <8 mitoses/2 mm^2^; M2 =  medium if 8–14 mitoses/2 mm^2^; and M3 =  high if 15 or more mitoses/2 mm^2^ [11].

### 2.4 Immunohistochemistry

Tissue microarrays (TMAs) were constructed using three replicate 0.6 mm cores from different invasive regions for each tumor, and four µm thick sections were cut from the TMA blocks. Immunohistochemical staining for Bcl2 (dilution 1∶200, code MO887, DAKO, clone 124) was performed using the Bond automated staining machine (Leica, Germany) with the Bond polymer refine detection kit (Leica, cat. no DS9800): peroxidase block 5 min, antigen retrieval with epitope retrieval 1, 20 min 99°, primary antibody 15 min RT, Bond polymer 8 min RT, DAB 10 min RT. Lymphoid tissue was used as positive control and kidney tissue as negative control throughout the immunohistochemical stainings. Immunohistochemical data of two patients was lost in the process due to poor core morphology.

**Figure 1:**
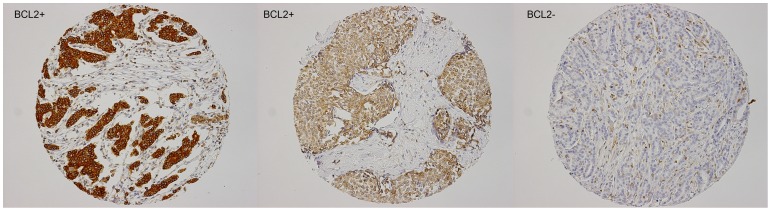
Expression of Bcl2 in male breast cancer.

Bcl2 was scored using TMAs by two experienced observers as negative when less than 10% of tumor cells showed expression, otherwise Bcl2 was scored as positive (fig. 1) [12], [21]. No difference in score was noticed when using the highest score between cores versus the mean of the three cores for each case.

The mitotic index/Bcl2 profiles were defined as M1/Bcl2+, M1/Bcl2−, M2/Bcl2+, M2/Bcl2−, M3/Bcl2+ and M3/Bcl2−.

### 2.5 Statistics

Information regarding prognosis and therapy was requested from the Integral Cancer Registration of The Netherlands (IKNL). Survival data was available for 103 (68%) cases. The mean follow up was 5.7 years (range 0.1–20.3 years). Therefore, survival analysis was based on 5 years survival rates.

Data analysis was performed using IBM SPSS statistics (version 20). Pearson’s chi-square or Fisher exact tests were used when appropriate. Survival rates were estimated using the Kaplan–Meier method and differences between curves were tested for significance using the log-rank test. Cox regression analysis was used for multivariate survival analysis. Hazard ratios (HRs) and 95% confidence intervals (95% CIs) were estimated for each variable. All tests were two-sided with a 95% CI and *p-*values less than 0.05 were considered significant.

## Results

### 3.1 Patients and Tumor Characteristics

The age of these patients ranged from 32 to 89 years (average: 66 years). Tumor size ranged from 0.2 to 7.2 cm (average: 2.235 cm). In 82.1% of patients their lymph node status was known by axillary lymph node dissection or sentinel node procedure and in 54.8% of these patients lymph node metastases were found. The majority of cases were diagnosed as invasive ductal carcinoma (90.1%). The remaining cases were lobular (n = 3), mixed type (ductal/lobular) (n = 3), invasive cribriform (n = 3), papillary (n = 2), mucinous (n = .2), invasive micropapillary (n = 1) or adenoid cystic carcinomas (n = 1).

Most tumors were ER positive (135/150; 90%) while PR and AR positivity was also common; this was 66% and 80% respectively. HER2 amplification was rare (5/150; 3.3%).

According to the modified Bloom and Richardson score 25.2% of the tumors were grade 1, 43.7% were grade 2 and 31.1% were grade 3.

### 3.2 Clinicopathological Significance of Bcl2 Expression

The majority of the tumors (140/149, 94%) showed positive Bcl2 expression using the 10% threshold, and 92% of the tumors (137/149) showed positive Bcl2 expression when applying a 30% threshold. Bcl2 expression had no significant association with favorable clinicopathologic features such as small tumor size, low mitotic count and low grade (Table 2), irrespective of these thresholds.

**Table 2:** Associations between Bcl-2 expression and clinicopathologic features in male breast cancer.

	*n (% of total)*	*Bcl2* −	*Bcl2* +	P*-value*
Size T				
T1	77; 53.8%	5; 7.7%	72; 92.3%	*0.232*
				
T2	64; 44.1%	3; 4.7%	61; 95.3%	
				
T3	4; 2.8%	1; 25.0%	3; 75.0%	
				
Grade				
G1	38; 25.3%	1; 2.6%	37; 97.4%	*0.618*
				
G2	65; 44.0%	5; 9.1%	60; 90.9%	
				
G3	46; 30.7%	3; 6.5%	43; 93.5%	
				
Mitosis				
M1	60; 40.0%	3; 5.0%	57; 95.0%	*0.908*
				
M2	38; 26.0%	3; 10.3%	35; 89.7%	
				
M3	51; 34.0%	3; 5.9%	48; 94.1%	
				

There was a positive correlation between Bcl2 and ER status (p = 0.04). No significant correlation was found between Bcl2 expression and p53 or HER2 expression.

### 3.3 Survival Analysis

**Figure 2:**
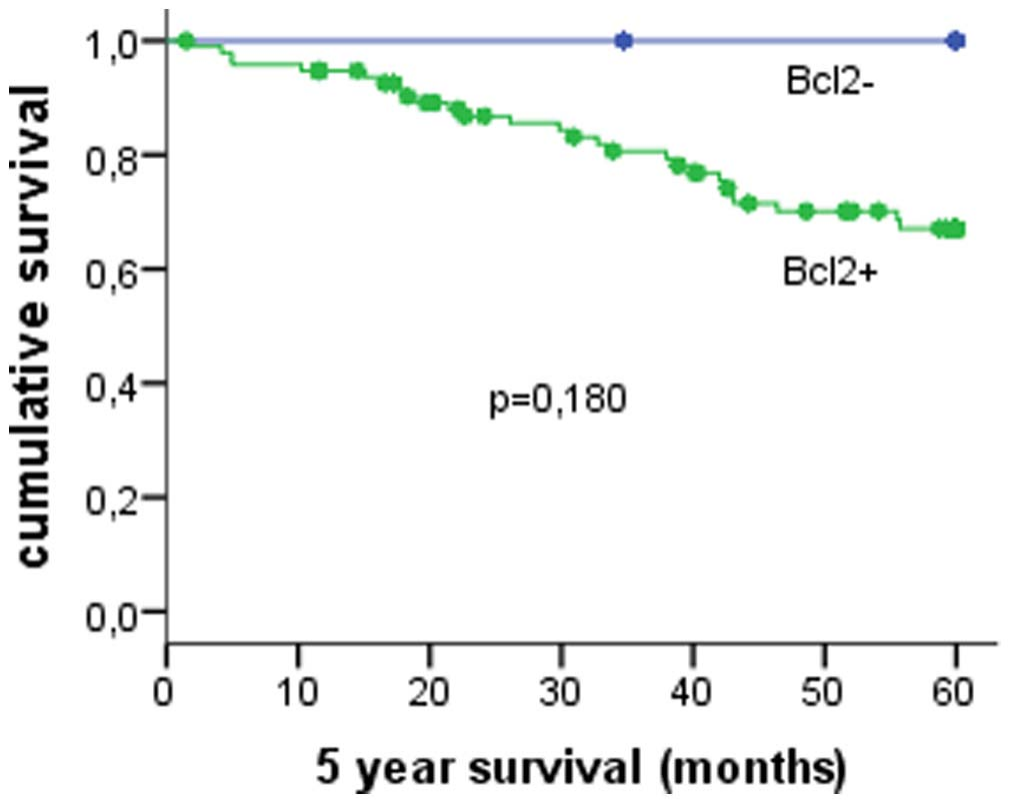
Survival curves for 103 male breast cancer cases with low or high Bcl2 expression.

Bcl2 expression was assessed in 101/103 cases with available survival data. In univariate survival analysis, Bcl2 expression was not associated with survival (p = 0.180)(fig. 2), as was true for the Mitotic Index (M1–M2–M3) (p = 0.144) (Table 3).

**Table 3:** Survival analysis results of 151 male breast cancers.

Feature	Grouping	5-Year survival			
		N	Alive	Survival rate (%)	P-value
Tumor size	T1	54	44	81.5	0.007*
	T2	44	29	65.9	
	T3	2	0	0.0	
					
Mitoses/2 mm^2^	0–7	43	36	83.7	0.144
	8–14	30	20	66.7	
	15>	30	19	63.3	
					
Grade	I	25	22	88.0	0.026*
	II	50	38	76.0	
	III	28	15	53.6	
Lymph node metastases	Absent	43	36	83.7	0.128
	Present	48	35	72.9	
					
Bcl2	Positive	96	69	71.9	0.180
	Negative	5	5	100	
					
M/Bcl2 profiles	M1/Bcl2+	42	35	83.3	0.262
	M1/Bcl2−	1	1	100	
	M2/Bcl2+	26	17	65.4	
	M2/Bcl2−	2	2	100	
	M3/Bcl2+	28	17	60.7	
	M3/Bcl2−	2	2	100	

The *asterisk* indicates a statistically significant association (p < 0.05) with survival.

In multivariate Cox regression only tumor size and histological grade emerged as independent prognostic factors. None of the other features had additional prognostic value.

**Figure 3:**
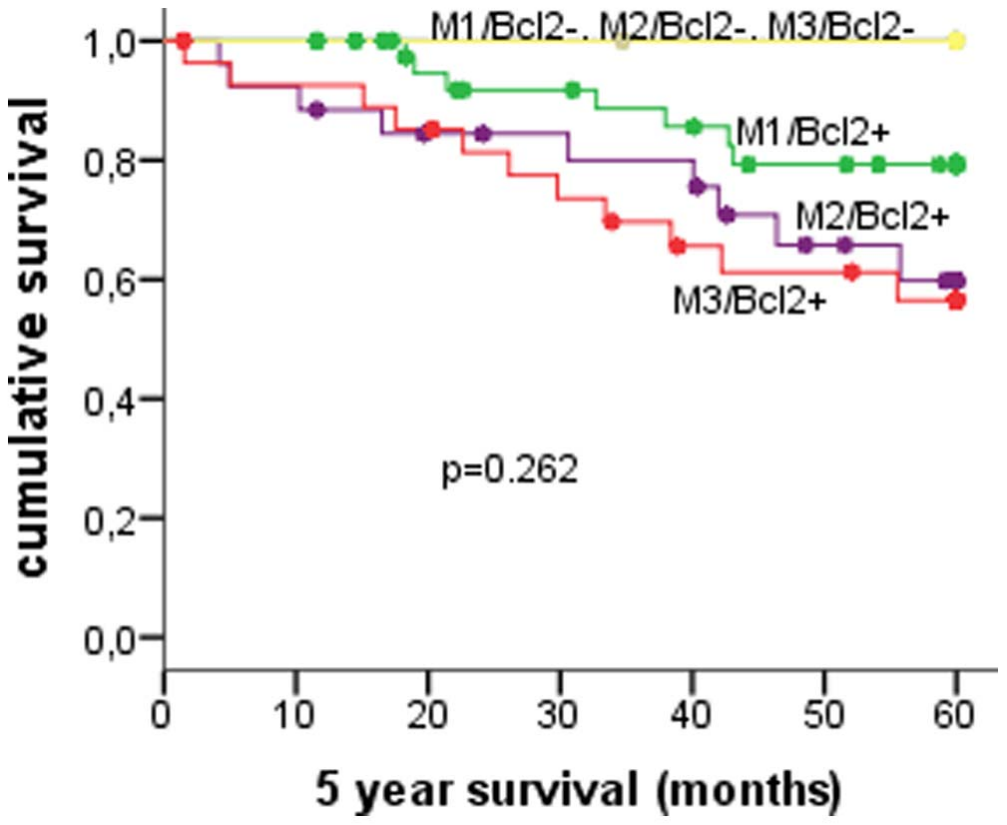
Survival curves for 103 male breast cancer cases according to different classes of the mitotic (M1–M2–M3)-Bcl2 expression grouping previously described in female breast cancer.

The combination of Bcl2 expression with mitotic index as described before in FBC did not predict survival in MBC (n = 101) (Table 3) (fig. 3).

## Discussion

The aim of the present study was to investigate the prognostic value of Bcl2 in conjunction with mitotic index in MBC, a combination previously described to have strong prognostic value in FBC. The vast majority of MBC showed Bcl2 expression, but Bcl2 expression had no significant associations with clinicopathologic features such as tumor size, mitotic count and grade. Although Bcl2 negative cases appear to have a better prognosis than Bcl2 positive cases this finding was not significant as only a few (5/101) cases were Bcl2 negative. In univariate and multivariate survival analysis, Bcl2 had no prognostic value. The Bcl2/mitotic index combination as previously described in FBC as a useful prognosticator, did not predict survival of MBC either. The widespread Bcl2 expression as demonstrated in the present study is largely in accordance with the few previous studies on Bcl2 in MBC [2], [13], [14], [15], although the percentages of positive cases vary somewhat in these studies due to variations in methodology and thresholds (Table 4).

**Table 4:** Overview of studies on Bcl2 in male breast cancer.

Article	n	Cutoff		% positive	Associations
Weber Chapius et al. Eur J Cancer. 1996	66	30%	67%		inverse association with p53, no correlation with survival
Rayson et al. Cancer. 1998	77	20%	94%		unable to assess prognostic value
Pich et al. Virchows Arch. 1998	34	30%	82.3%		trend for association with low tumor stage, no association with survival
Pich et al. J Clin Oncol. 2000	50	30%	74%		no prognostic value
Temmim et al. The Breast. 2001	18	any+	78%		inverse association with p53 and Ki67
Present study	151	10%	94%		no prognostic value
		30%	92%		no prognostic value

The biological mechanisms of Bcl2 as a prognostic factor for breast cancer remain largely unclear. The Bcl2 (B-cell CLL/lymphoma 2) gene is located in 18q21.33 and encodes an integral outer mitochondrial membrane protein that blocks the apoptotic death of some cells. Bcl2 is well known as an anti-apoptotic oncogene in lymphoma [22], however the paradoxical function of the tumor suppressor gene has been reported in many solid tumors, including breast cancer [23], [24]. Bcl2 may be both oncogenic and tumor suppressive in specific cell types or under specific conditions, and it is postulated that the tumor suppressive effect is more prominent in breast cancer. Bcl2 expression has extensively been studied in FBC [12], [21], [25]–[30], rather consistently showing its prognostic value also independent of hormone receptor status. A correlation between Bcl2 expression and hormonal receptor status has been repetitively reported in FBC [31], [32]. Bcl2 was supposed to be up-regulated by oestrogen, possibly as a result of direct transcriptional induction with negative regulation by p53-dependent mechanisms [21]. In the present study we find a positive correlation between Bcl2 and ER status (p = 0.04), but not as strong as described in FBC (p<0.00001) [31], [32]. On the other hand we did not find a significant inverse relationship between Bcl2 expression and p53 accumulation. In literature data concerning the relationship between Bcl2 and ER/p53 in MBC are not consistent, making it difficult to draw conclusions from these findings [2], [13], [14].

Compared to FBC, there is a higher expression of Bcl2 (94% vs. 68.2%) in MBC [25]. Its expression was not associated with favorable clinicopathologic features such as small tumor size, low mitotic count and low grade in male breast cancer (Table 2), which is largely in line with previous studies (Table 4). None of the tumors expressing HER2, which has been previously reported to be associated with more aggressive phenotype and adverse prognosis in MBC [33], showed Bcl2 expression. However, we found no significant association between HER2 and Bcl2 expression, which can be due to the fact that a very low percentage (3.3%) of the tumors expressed HER2. Only a few Bcl2 studies have been performed in small cohorts of MBC [2], [13], [14], [15], showing no relation with survival (Table 4). Also the present study (n = 151) failed to show prognostic value of Bcl2 expression as a single feature, nor did it emerge in multivariate survival analysis. In FBC, a combination of Bcl2 expression and mitotic index (grouped according to the Nottingham grading classes) appeared to have very strong prognostic value in a large cohort of 1650 patients [12]. Other than in FBC, that very combination of mitotic index and Bcl2 expression had no prognostic value in MBC. This is not surprising given the fact that in our study we also found no significant associations between the M1–M2–M3 mitotic index groups and survival, although a mitotic index threshold of 8 per 2 mm^2^ did have strong prognostic value in our previous study [34]. Apparently, MBC and FBC differ with regard to the clinical significance of Bcl2 expression and the prognostically optimal thresholds for mitotic index. The biological background for this is unclear until now.

In conclusion, Bcl2 expression is common in MBC, but is not associated with major clinicopathologic features and does not seem to have prognostic value, also not when combined with mitotic index, which previously did seem to work well in FBC.
